# Mosquito Infection Responses to Developing Filarial Worms

**DOI:** 10.1371/journal.pntd.0000529

**Published:** 2009-10-13

**Authors:** Sara M. Erickson, Zhiyong Xi, George F. Mayhew, Jose L. Ramirez, Matthew T. Aliota, Bruce M. Christensen, George Dimopoulos

**Affiliations:** 1 Department of Pathobiological Sciences, University of Wisconsin-Madison, Madison, Wisconsin, United States of America; 2 W. Harry Feinstone Department of Molecular Microbiology and Immunology, Bloomberg School of Public Health, Johns Hopkins University, Baltimore, Maryland, United States of America; Yale School of Public Health, United States of America

## Abstract

Human lymphatic filariasis is a mosquito-vectored disease caused by the nematode parasites *Wuchereria bancrofti*, *Brugia malayi* and *Brugia timori*. These are relatively large roundworms that can cause considerable damage in compatible mosquito vectors. In order to assess how mosquitoes respond to infection in compatible mosquito-filarial worm associations, microarray analysis was used to evaluate transcriptome changes in *Aedes aegypti* at various times during *B. malayi* development. Changes in transcript abundance in response to the different stages of *B. malayi* infection were diverse. At the early stages of midgut and thoracic muscle cell penetration, a greater number of genes were repressed compared to those that were induced (20 vs. 8). The non-feeding, intracellular first-stage larvae elicited few differences, with 4 transcripts showing an increased and 9 a decreased abundance relative to controls. Several cecropin transcripts increased in abundance after parasites molted to second-stage larvae. However, the greatest number of transcripts changed in abundance after larvae molted to third-stage larvae and migrated to the head and proboscis (120 induced, 38 repressed), including a large number of putative, immunity-related genes (∼13% of genes with predicted functions). To test whether the innate immune system of mosquitoes was capable of modulating permissiveness to the parasite, we activated the Toll and Imd pathway controlled rel family transcription factors Rel1 and Rel2 (by RNA interference knockdown of the pathway's negative regulators Cactus and Caspar) during the early stages of infection with *B. malayi*. The activation of either of these immune signaling pathways, or knockdown of the Toll pathway, did not affect *B. malayi* in *Ae. aegypti*. The possibility of LF parasites evading mosquito immune responses during successful development is discussed.

## Author Summary

Filarial worms that cause human lymphatic filariasis (LF) are transmitted by many species of mosquitoes. Within susceptible mosquitoes, *Brugia malayi* develop from microfilariae (mf) to infective-stage larvae (L3s), in approximately eight days. These nematodes develop as intracellular parasites within mosquito flight muscle cells, in which they ingest cellular material and eventually cause cell death when L3s migrate to the mosquito's proboscis. We examined the effects of *B. malayi* parasitism on *Aedes aegypti* by analyzing changes in mosquito gene expression at different stages of parasite development. We found that a few genes were differentially expressed at the RNA level relative to non-infected controls. The majority of changes occurred at two time periods, when the filarial worms began feeding and when the L3s were in the head and proboscis. Many transcriptional changes in the later group concur with documented descriptions of tissue damage, clean-up and repair that occurs in mosquitoes infected with filarial worms. In addition, we activated two innate immunity signaling pathways and observed the effects on filarial worm development. *B. malayi* seems to be capable of evading these immune responses, because its development was not impeded by the activation of either the Toll or Imd signal pathways in *Ae. aegypti*.

## Introduction

It is estimated that 120 million people are infected with *Wuchereria bancrofti*, *Brugia malayi*, or *B. timori*, the mosquito-transmitted, parasitic nematodes that cause human lymphatic filariasis (LF). In approximately 40% of cases, the disease is manifested by lymphedema of the extremities or hydrocoele. Although human LF does not increase mortality in endemic areas, morbidity causes major economic losses and often leads to psychosocial and psychosexual conditions in infected individuals [1]. Recent efforts by the Global Program for the Elimination of Lymphatic Filariasis (GPELF) have decreased the numbers of individuals infected with, and at risk for, this parasitic disease [2].

Several different mosquito species within the genera *Culex*, *Anopheles*, *Aedes* and *Mansonia* can serve as primary vectors of LF parasites. The geographical location and habitat type influence which mosquito species function as vectors in any particular endemic area. Biological transmission of filarial worms is termed cyclodevelopmental, i.e., the parasite undergoes development within the vector to become infective to the vertebrate host, but does not multiply. In competent vectors, microfilariae (mf), produced by adult female worms and found circulating in the peripheral blood, are ingested with a blood meal and will quickly (within 2 hr) penetrate the midgut epithelium to access the hemocoel [3]. Mf migrate in the mosquito's hemolymph to reach the thoracic musculature and from there penetrate into the indirect flight muscles. This tissue is the site of development, where mf undergo two molts and emerge as infective-stage larvae (L3s). Approximately eight days after exposure, L3s migrate to the head and proboscis from where they escape by penetrating the labellum of the proboscis when the mosquito takes a blood meal. Within the human host, the parasites undergo two additional molts and grow as they migrate to lymphatic vessels where adult male and female worms mate and females give birth to mf. Mf then make their way into the circulating blood from where they can be ingested by another blood feeding mosquito.

LF parasites grow nearly seven times in length (*B. malayi* grow from ∼200 to ∼1,350 µm in length, from mf to L3s respectively) during the extrinsic developmental period within the mosquito [4]. As parasites develop, the mosquito must tolerate a series of insults due to parasite activities, e.g., migrating mf damage both midgut [5] and muscle cells as they penetrate through or into them; second-stage larvae (L2s) actively ingest mosquito cellular components; L3s are very large and migrate out of the thoracic muscles and through the body cavity to reach the head and proboscis. Ultrastructural studies of *Aedes* mosquitoes infected with *Brugia* parasites have revealed that nuclear enlargement (a sign of a putative repair response) occurs in both *Brugia*-infected and neighboring non-infected muscle cells, and that complete degeneration of infected muscle cells occurs once L3s exit the flight muscles [6]. Other studies have shown that mosquito flight muscle cells become devoid of glycogen granules following infection with *Brugia* parasites [7],[8]. Considering the amount of tissue damage observed in muscle cells, it is not surprising that *Brugia*-infected mosquitoes are known to have decreased flight activity and longevity [9],[10]. But the successful development (and subsequent transmission) of LF parasites depends on the ability of competent mosquito vectors to survive infection.

Some mosquitoes are able to limit or prevent filarial worm infections with various refractory or resistance mechanisms. For example, mf can be damaged during ingestion by an armed pharyngeal and/or cibarial pump (often found in *Anopheles* spp.), inhibiting them from penetrating the midgut wall [11],[12]. Mf that successfully penetrate the midgut and enter the hemocoel come in contact with hemolymph components, including circulating blood cells called hemocytes. Melanotic encapsulation is a hemocyte-mediated, innate immune response that can be very specific and robust, and can limit or prevent parasite development in some mosquito species [13]. In contrast, the involvement of a humoral immune response is not well understood in many compatible filarial worm-mosquito systems. Some parasites are able to evade or suppress a host's immune system in order to survive, but it is unknown if such interactions occur between LF parasites and mosquitoes [14].

Previous studies have investigated the effect of an activated mosquito immune response on filarial worm development, but the results remain inconclusive. When bacteria are inoculated into the mosquito hemocoel, to induce the expression of infection responsive immune factors prior to filarial worm exposure, a reduced *B. malayi* prevalence and mean intensity in *Ae. aegypti* was observed as compared to non-inoculated controls [15]. However, when the same bacterial strains and inoculation procedures were used to pre-activate the immune response of *Culex pipiens* prior to *W. bancrofti* exposure, there was no difference in prevalence or mean intensity between bacteria-inoculated and control mosquitoes [16]. Further investigation is needed to assess the effects of an activated mosquito immune response on a LF parasite infection.

The role of Toll and Imd signaling pathways in the immune recognition, modulation, and response of mosquitoes to LF parasites has yet to be examined. Recently, Xi *et al.* [17] developed methods to manipulate these immune signaling pathways in *Ae. aegypti* by (1) gene silencing of Cactus, a negative regulator of the Toll pathway, (2) gene silencing of MyD88, an adaptor required for endogenous Toll pathway signal transduction, and (3) gene silencing of Caspar, a negative regulator of the Imd pathway. Using these tools, the Toll and Imd pathways are transiently relieved from endogenous suppression (1 and 3 above) or made unresponsive to detected stimuli (2 above).

In this study we assess transcriptome changes associated with the development of *B. malayi* in *Ae. aegypti* and investigate the effect of the immune signaling pathways, Toll and Imd, on parasite development.

## Materials and Methods

All animals were handled in strict accordance with good animal practice as defined by the University of Wisconsin-Madison School of Veterinary Medicine/University of Wisconsin Research Animal Resource Center, and all animal work was approved by these entities.

### Mosquito Maintenance

*Aedes aegypti* black-eyed, Liverpool (LVP) strain used in this study were maintained at the University of Wisconsin-Madison as previously described [18]. Briefly, mosquitoes were maintained on 0.3 M sucrose in an environmental chamber at 26.5±1°C, 75±10% RH, and with a 16 hr light and 8 hr dark photoperiod with a 90 minute crepuscular period at the beginning and end of each light period. *Ae. aegypti* LVP was originally selected for susceptibility to *Brugia malayi* by Macdonald in 1962. This strain supports the development of mf to L3s. Four- to five-day-old mosquitoes were sucrose starved for 14 to 16 hours prior to blood feeding.

### *Brugia malayi* Infections

Mosquitoes were exposed to *B. malayi* (originally obtained from the University of Georgia NIH/NIAD Filariasis Research Reagent Repository Center) by feeding on ketamine/xylazine anesthetized, dark-clawed Mongolian gerbils, *Meriones unguiculatus*. The same animals were used for all three biological replicates. Microfilaremias were determined, using blood from orbital punctures, immediately before each feeding and ranged from 50–150 mf per 20 µl of blood. Control mosquitoes were exposed to anesthetized, uninfected gerbils. Mosquitoes that fed to repletion were separated into cartons and maintained on 0.3 M sucrose in the laboratory.

### Mosquito Dissections

In early stages of development (1 h to 3 d post-infection [PI]), individual mosquitoes were separated into midgut, thorax and abdomen (with midgut removed) and dissected in *Aedes* saline [19]. Tissue dissections were cover-slipped and parasites were observed with a compound microscope using phase-contrast optics. The same procedure was used for dissections at 5–6 d PI, except only thoraces were dissected and examined. At 8–9 d PI, the thorax was processed as described above, but the abdomen and head & proboscis were dissected in separate drops of *Aedes* saline to observe L3s using a dissection microscope. Individual mosquitoes were dissected in a drop of *Aedes* saline for the recovery of L3s at 13–14 d PI. Images of *B. malayi* developmental stages were captured and processed as previously described, with the addition of Nomarski optics [20].

### Mosquito Collection

Five sample groups were created to study the transcriptional response of mosquitoes to *B. malayi* development. In each group, 20 mosquitoes were collected for RNA extraction. These sample groups are defined by the time after the blood meal and represent significantly different stages of parasite development. Briefly, Group 1 consisted of mosquitoes collected at 1, 6, 12 and 24 h PI. At these early time points, mf are penetrating the mosquito midgut, migrating through the hemocoel and penetrating thoracic muscle cells. Group 2 was collected at 2–3 d PI, a time when mf have differentiated into intracellular first-stage larvae (L1s). At 5–6 d PI, *B. malayi* complete the molt to second-stage larvae (L2s) and actively feed on mosquito muscle tissue (Group 3). In Group 4, at 8–9 d PI, parasite development is complete with a second molt to the L3s. Tissue damage continues as L3s break out of the thoracic muscles and migrate to the mosquito's head and proboscis. The final collection (Group 5), made at 13–14 d PI, occurs when the majority of L3s are located in the head and proboscis (see [4],[21]). Five mosquitoes, from both *B. malayi*-infected and uninfected blood meals, were collected at 1, 6, 12, and 24 h PI and ten mosquitoes at 2, 3, 5, 6, 8, 9, 13 and 14 d PI for transcriptional analysis. Mosquitoes were pooled (5 mosquitoes/tube), flash frozen in liquid nitrogen and stored at −80°C prior to total RNA extraction using RNeasy (QIAGEN). In addition, five *B. malayi*-infected mosquitoes were dissected to verify filarial worm infection and to determine the stage of parasite development at each time point. Three biological replications were completed.

### Microarray Procedures and Analysis

Microarray assays were conducted and analyzed as reported previously [17],[22]. A full genome microarray platform (Agilent; 4×44k) was used with the probe sequences identical to the previous version (1×22k) [22]. In brief, 2–3 µg total RNA was used for probe synthesis of Cy3- and Cy5-labeled dCTP. Hybridizations were conducted with an Agilent Technologies *In Situ* Hybridization kit at 60°C according to the manufacturer's instructions. Three independent biological replicate assays were performed. Hybridization intensities were determined with an Axon GenePix 4200AL scanner, and images were analyzed with Gene Pix software. To produce the expression data, the background-subtracted median fluorescent values were normalized according to a LOWESS normalization method to reduce dye-specific biases, and Cy5/Cy3 ratios from replicate assays were subjected to *t*-tests at a significance level of p<0.05 using TIGR MIDAS and MeV software [23]. Expression data from all replicate assays were averaged with the GEPAS microarray preprocessing software prior to logarithm (base 2) transformation. Self-self hybridizations have been used to determine the cut-off value for the significance of gene abundance on these microarrays to 0.8 in log_2_ scale, which corresponds to 1.74- fold regulation [22]. For genes with P<0.05, the average ratio was used as the final fold change; for genes with P>0.05, the inconsistent probes (with distance to the median of replicate probe ratios larger than 0.8 log_2_) were removed, and only the value from a gene with at least two replicates was further averaged. The robustness of these microarray gene expression assays were validated through qPCR (Text S1).

### Real-Time PCR Assays

Real time PCR assays were conducted as previously described to validate gene silencing efficiency and microarray expression data for selected genes [24]. Briefly, RNA samples were treated with Turbo DNAse (Ambion, Austin, Texas, United States) and reverse-transcribed using Superscript III (Invitrogen, Carlsbad, California, United States) with random hexamers. Transcript relative quantification was performed using the QuantiTect SYBR Green PCR kit (Qiagen) and ABI Detection System ABI Prism 7300 (Applied Biosystems, Foster City, California, United States). qRT-PCR reactions were conducted using a 10 minute step at 94°C and 40 cycles of 15 seconds at 94°C, 15 seconds at 55°C and 15 seconds at 72°C. Three independent biological replicates were conducted and all PCR reactions were performed in triplicate. Transcript abundance was normalized against the ribosomal protein S7 gene. All primers used for qPCR assay are presented in Table S1. The primer sequences used to verify gene knockdown efficiency included: S7 (AAEL009496-RA) forward: 5′-GGGACAAATCGGCCAGGCTATC-3′, reverse: 5′- TCGTGGACGCTTCTGCTTGTTG-3′; Caspar (AAEL003579-RA) forward: 5′-GAATCCGAGCGAGCCGATGC-3′, reverse: 5′-CGTAGTCCAGCGTTGTGAGGTC-3′; Cactus (AAEL000709-RA) forward: 5′-AGACAGCCGCACCTTCGATTCC-3′, reverse: 5′-CGCTTCGGTAGCCTCGTGGATC-3′; MyD88 (AAEL007768) forward: 5′-CATCCCATTCAGTTTCTCAGC-3′, reverse: 5′-ACCGGTTGGAAGTTCTGATG-3′. A complete list of PCR primer sequences is presented in Table S1.

### Gene Silencing Assays

RNAi was conducted by intrathoracic injection of dsRNA using described methodology [24],[25]. Mosquitoes were three to four days old at the time of blood feeding and dsRNA was injected either 48 h before or after parasite exposure, i.e., dsRNA injections were performed on non-blood fed one- to two-day-old mosquitoes and on blood fed, five- to six-day-old mosquitoes. Approximately 0.5 µl of dsRNAs (1.0 or 0.5 µg/µl) were injected into the thorax of cold-anesthetized mosquitoes. The primers used to synthesize Cactus, Caspar and MyD88 dsRNA have been published previously [17]. To synthesize GFP dsRNA, methods described by Bartholomay *et al.* [25] were used with minor changes. The following sequences were annealed by heating at 95°C for 5 min and slow cooling: GFP_F 5′-TAGTACAACTACAACAGCCACAACGTCTATATCATGGCCGACAAGCAGA AGAACGGCATCAAGGTGAACTTCAAGATCCGCCACAACA-3′ and GFP_R: 5′-TCGATGTTGTGGCGGATCTTGAAGTTCACCTTGATGCCGTTCTTCTGCTTGTCGGCCATGATATAGACGTTGTGGCTGTTGTAGTTGTA-3′ (Integrated DNA Technologies, Inc., Coralville, IA). This dsDNA was ligated into pBlueScript KS+ (Stratagene) at XbaI (T7) and SalI (T3) sites. A complete list of PCR primer sequences is presented in Table S1.

### Infection

*B. malayi* exposures were performed as described above and microfilaremias ranged from 35–162 mf per 20 µl of blood. Mosquitoes injected with GFP dsRNA were blood fed on each infected gerbil and used as a control for parasite infections. Mosquito mortality was observed every 24 h and mosquitoes were dissected at 6 d or 12–13 d PI to observe parasite development. To verify gene knockdown, five mosquitoes were collected 48 h post dsRNA injection, placed in a microcentrifuge tube, flash frozen and stored at −80°C until RNA extraction. Real-time PCR was used to quantify gene silencing efficiency. Silencing of Cactus, Caspar and MyD88 resulted in a reduction of mRNA levels by 60%, 84% and 27%, respectively. For each exposure, the prevalence and mean intensity of infection was calculated. Comparisons of mean intensities and mosquito mortality curves were done with the Mann-Whitney and Log-Rank tests, respectively, using GraphPad Prism 5 (GraphPad Software, Inc., La Jolla, CA). Results were considered significant at P≤0.05.

## Results

### *Brugia malayi* Development in *Aedes aegypti*

**Figure 1:**
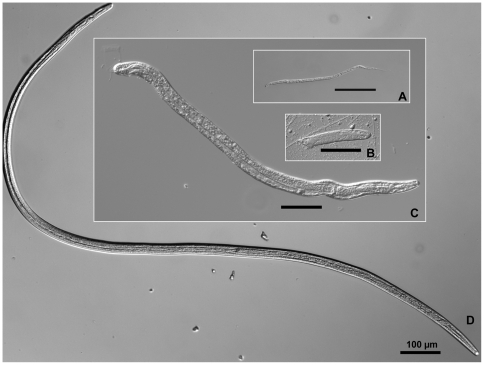
Relative sizes of *Brugia malayi* developmental stages that occur within compatible mosquito hosts: An example of cyclo-developmental transmission. A. microfilariae are ingested during blood feeding; B. parasites differentiate into non-feeding, first-stage larvae within mosquito indirect flight muscle cells; C. following the first molt, second-stage larvae remain intracellular parasites which ingest cellular material into their newly developed digestive tract. D. third-stage larvae leave the muscle cells and migrate to the mosquito's head and proboscis where they will exit through the mosquito cuticle during blood feeding.

The development of *B. malayi* was observed at 1 h to 14 d PI for each biological replicate and is summarized in Table S2. Worms were recovered from 166 of the 180 mosquitoes examined for an overall infection prevalence of 92%. Mf were recovered from 1 h to 3 d PI, but after 24 h PI mf were no longer the most abundant developmental stage recovered. At 2 d PI, mf recovery began to decrease and almost all worms had differentiated into intracellular L1s by 3 d PI. Parasites molted to L2s in the thoracic musculature by 5 d PI, and were the only developmental stage identified in transcriptional group 3. The molt from L2 to L3 occurred at 8–9 d PI. At 8 d PI, L2s and L3s were recovered in the thorax, and only 4% of the worms were located in the head and proboscis. In contrast, at 9 d PI, both L2s and L3s were observed in the thorax region, but the majority were L3s and 47% of all recovered worms were located in the head and proboscis. By 13–14 d PI, all parasites had developed into L3s. Images of *B. malayi* development, from mf to L3s, are presented in Figure 1. The prevalence of L3s (for all three biological replicates at 13–14 d PI) was 80% (n = 30) and the mean intensity was 6.9±5.7.

### Global Response of *Ae. aegypti* to *B. malayi* Infection

**Figure 2:**
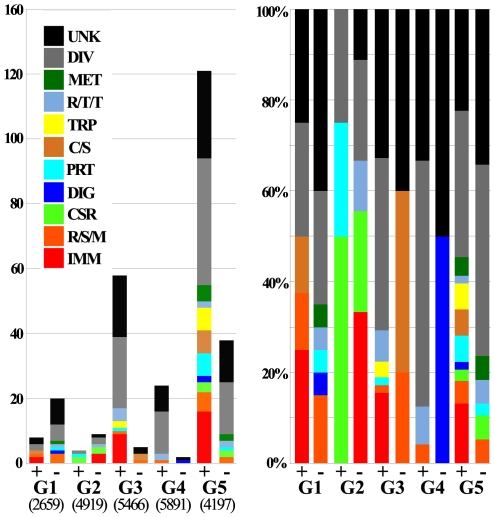
Global transcriptional analysis of the response of a compatible mosquito infected with successfully developing *Brugia malayi*. A. Regulated (differentially expressed above a 1.7-fold threshold) genes for each experimental group. (+) indicates up-regulated in infected mosquitoes and (−) indicates down-regulated. Assayable genes (those that gave signal intensities above a standard cutoff threshold) are indicated below the graph. Functional group distributions are the same to those used in Nene *et al.*, 2007 and Xi *et al.*, 2008: IMM: immunity, R/S/M: redox, stress, mitochondrial, CSR: chemosensory reception, DIG: blood digestive, PRT: proteolysis, C/S: cytoskeletal, structural, TRP: transport, R/T/T: replication, transcription, translation, MET: metabolism, DIV: diverse, UKN: unknown. B. Percent distribution of functional group for each up- and down-regulated experimental group. Transcription data are presented in Table S5.

The *Ae. aegypti* global transcript responses to the successful development of *B. malayi* were determined using a genome microarray expression approach. These transcriptome infection-response patterns differed significantly, in both the number of regulated genes and their direction of regulation, at the different parasite development stages (Fig. 2A). A general suppression of transcription was evident during the early stages of infection (group 1); 20 genes were down-regulated while only 8 genes were up-regulated. However, this transcriptional suppression was reduced when parasites developed into L1s (group 2), and reversed when they became L2s (group 3); 59 genes were up-regulated and only 5 genes were down-regulated at this stage. Parasite infection at later stages of infection mainly caused transcriptional up-regulation (groups 4 and 5). Strikingly, 120 genes were up-regulated in group 5 that represented mosquitoes in which L3s had migrated to the head and proboscis from where they can be transmitted to a host upon blood feeding. As many as 158 mosquito genes were differentially expressed at this late stage. The second largest number of infection-responsive genes was observed when L2s were present (group 3).

Interestingly, the transcripts that were over represented in the groups that displayed the most prominent transcriptional regulation (3 and 5), were highly enriched with putative immune genes (Fig. 2). Specifically, several antimicrobial peptide effector genes were strongly induced in group 3 and a number of putative pattern recognition receptor and signal modulator genes were up-regulated in group 5 (Table 1). Group 5 also contained several serine protease cascade components and putative melanization –related factor transcripts in increased abundance (Tables 1 and 2).

**Table 1:** Cluster analysis of *Aedes aegypti* immunity genes transcribed by *Brugia malayi*-infected mosquitoes.

Cluster	Vectorbase ID	Gene Name	Relative Transcript Abundance^a^				
			Group 1 (Infection)	Group 2 (Non-feeding L1s)	Group 3 (Feeding L2s)	Group 4 (Molt to L3s)	Group 5 (Persistent L3s)
1	AAEL005748-RA	elastase, putative	1.70				
1	AAEL003857-RA	DEF D	1.94				
2	AAEL000598-RA	CEC D			1.85		
2	AAEL000611-RA	CEC E			3.04		
2	AAEL000625-RA	CEC F		−1.97	2.12		
2	AAEL000627-RA	CEC A		−1.89	2.43		
2	AAEL015515-RA	CEC G			1.99		
2	AAEL000621-RA	CEC N			2.01		
2	AAEL001929-RA	SPZ 5			2.11		
2	AAEL012958-RA	conserved hypothetical protein			1.89		
2	AAEL010606-RA	down syndrome cell adhesion molecule			1.77		
3	AAEL003294-RA	FREP					1.73
3	AAEL009178-RA	GNBPB4					1.68
3	AAEL014989-RA	peptidoglycan recognition protein-1, putative					2.02
3	AAEL007037-RA	PGRPS4					1.86
3	AAEL005374-RA	SCRB1					1.96
3	AAEL014349-RA	CLIPSP					1.72
3	AAEL005060-RA	CLIPSP					2.24
3	AAEL005644-RA	CLIPSPH					1.82
3	AAEL010267-RA	serine protease					1.72
3	AAEL009558-RA	serine protease, putative					1.80
3	AAEL010769-RA	SRPN6					1.74
3	AAEL004000-RA	TOLL10					1.75
3	AAEL002583-RA	TOLL7					1.70
3	AAEL013499-RA	PPO2					1.74

a Fold change, by Mosquito Transcriptional Group (Stage of Filarial Worm Development).

**Table 2:** Few *Aedes aegypti* genes are differentially transcribed in multiple stages of the infection response to *Brugia malayi*.

Vectorbase ID	Description	Function	Relative Transcript Abundance^a^				
			Group 1 (Infection)	Group 2 (Non-feeding L1s)	Group 3 (Feeding L2s)	Group 4 (Molt to L3s)	Group 5 (Persistent L3s)
AAEL002621-RA	hypothetical protein	DIV			2.02	1.83	
AAEL003014-RA	hypothetical protein	DIV			1.82	1.72	
AAEL003015-RA	protein phosphatase 2a, regulatory subunit	DIV			1.87	1.76	
AAEL004706-RA	conserved hypothetical protein	DIV		−1.92	2.56		
AAEL006167-RA	runt	DIV			2.10	1.73	
AAEL006567-RA	max binding protein, mnt	DIV			1.75	1.71	
AAEL008194-RA	protein phosphatase 2a, regulatory subunit	DIV			1.69	1.71	
AAEL009136-RA	hypothetical protein	DIV			1.87	1.92	
AAEL009417-RA	hypothetical protein	DIV		−1.75	2.42	1.87	
AAEL013349-RA	lethal(2)essential for life protein, l2efl	DIV			1.70	2.45	
AAEL000625-RA	CECF	IMM		−1.97	2.12		
AAEL000627-RA	CECA	IMM		−1.89	2.43		
AAEL015257-RA	hypothetical protein	PROT			1.86		1.73
AAEL013350-RA	heat shock protein 26 kD, putative	RSM			2.00	2.44	
AAEL001912-RA	forkhead protein/forkhead protein domain	RTT			1.96	1.82	
AAEL006944-RA	hypothetical protein	RTT			1.71	1.70	
AAEL007166-RA	hypothetical protein	RTT	−2.95	−2.04	2.30		
AAEL001392-RA	hypothetical protein	UNK		−3.06	1.79	1.98	
AAEL004149-RA	hypothetical protein	UNK			2.03	1.70	
AAEL005233-RA	hypothetical protein	UNK			2.28	1.69	
AAEL005265-RA	conserved hypothetical protein	UNK			1.91	1.73	
AAEL007108-RA	hypothetical protein	UNK			2.34	2.02	
AAEL009473-RA	conserved hypothetical protein	UNK			2.22	1.68	
AAEL011351-RA	hypothetical protein	UNK			−1.89	−2.02	
AAEL013178-RA	hypothetical protein	UNK			1.80		1.78
AAEL015259-RA	hypothetical protein	UNK	−1.82		1.75		

a Fold change, by Mosquito Transcriptional Group (Stage of Filarial Worm Development).

### Effect of the Toll and Imd Pathways on *B. malayi* Development

To investigate whether the mosquito's two major immune signaling pathways, Toll and Imd, had any effect on *B. malayi* infection we used an established gene silencing approach to either simulate the activation of the Toll and Imd pathway, by depleting their negative regulators Cactus and Caspar, respectively, or inhibit the Toll pathway through the depletion of the MyD88 factor [17],[26].

### Mosquito survival

**Figure 3:**
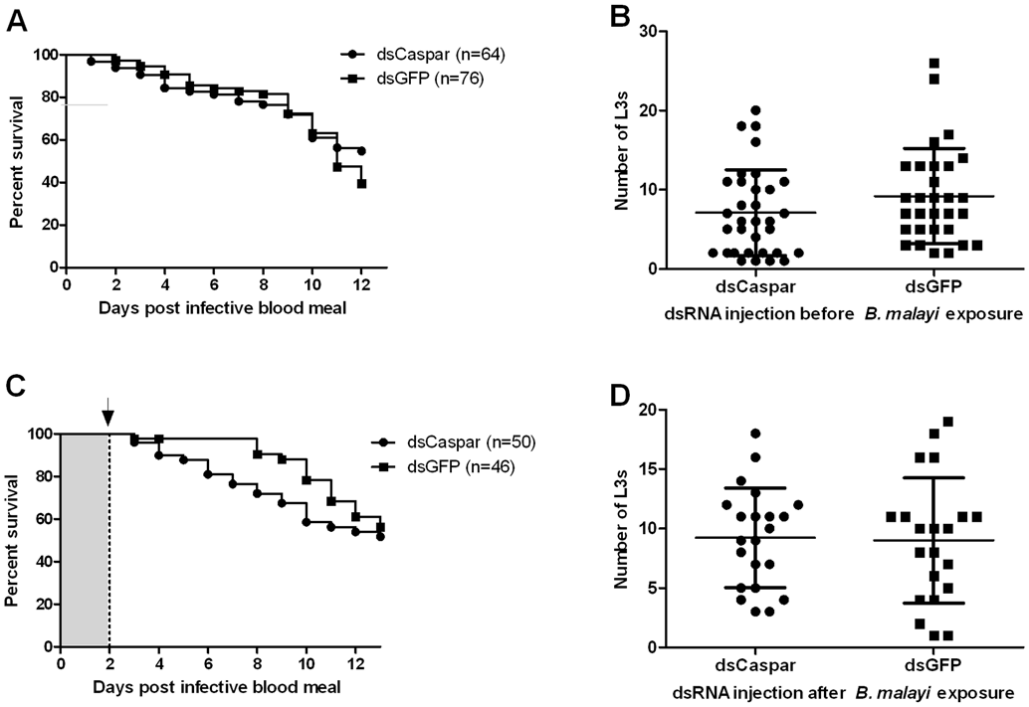
Gene knockdown of *Ae. aegypti* Caspar does not affect *B. malayi* development. A, B. Gene knockdown of *Ae. aegypti* Caspar at the time of parasite ingestion did not affect mosquito mortality (A; P = 0.23) or *B. malayi* development (B; P = 0.10), in comparison to control mosquitoes. C, D. Gene knockdown of *Ae. aegypti* Caspar at the time of the parasite's first molt (L1 to L2) did not affect mosquito mortality (C; P = 0.32) or *B. malayi* development (D; P = 0.72). Bars indicate the mean intensity (total number of L3s recovered per infected individual) and standard deviation (B and D). Log-rank and Mann-Whitney tests were used to compare mosquito mortality curves and parasite mean intensities, respectively. Arrow: dsRNA was injected intrathoracically two days following infective blood meal.

**Figure 4 pntd-0000529-g004:**
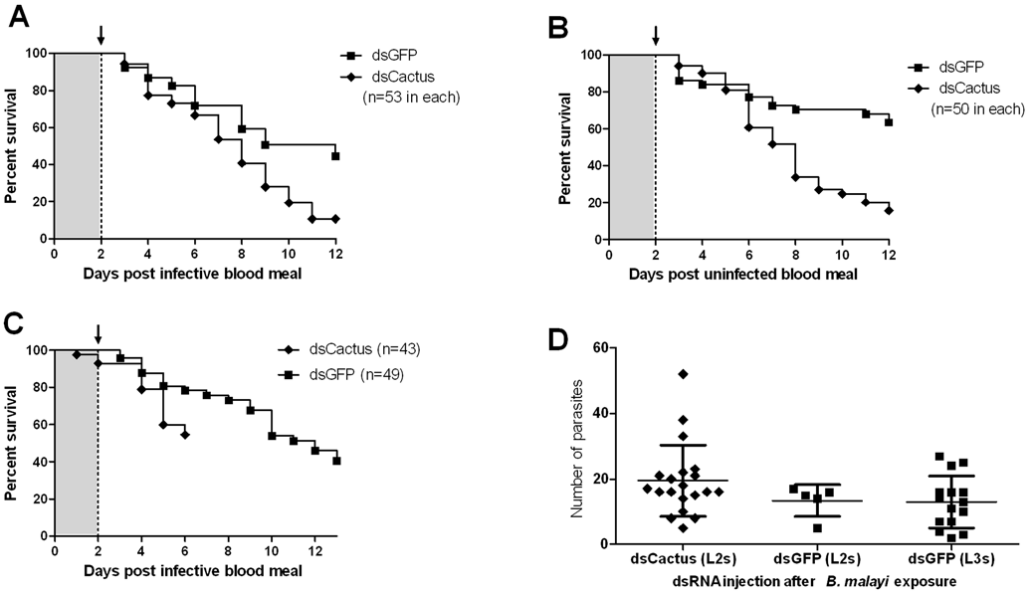
Gene knockdown of *Ae. aegypti* MyD88 does not affect *B. malayi* development. A, B. Gene knockdown of *Ae. aegypti* MyD88 during parasite ingestion did not affect mosquito mortality (A; P = 0.94) or *B. malayi* development (B; P = 0.14), compared to control mosquitoes. C, D. Knockdown of *Ae. aegypti* MyD88 when parasites first molt (L1 to L2) did not affect mosquito mortality (C; P = 0.30) or *B. malayi* development (D; P = 0.77). Bars indicate the mean intensity (total number of L3s recovered per infected individual) and standard deviation (B and D). Log-rank and Mann-Whitney tests were used to compare mosquito mortality curves and parasite mean intensities, respectively. Arrow: dsRNA was injected intrathoracically two days following infective blood meal.

**Figure 5:**
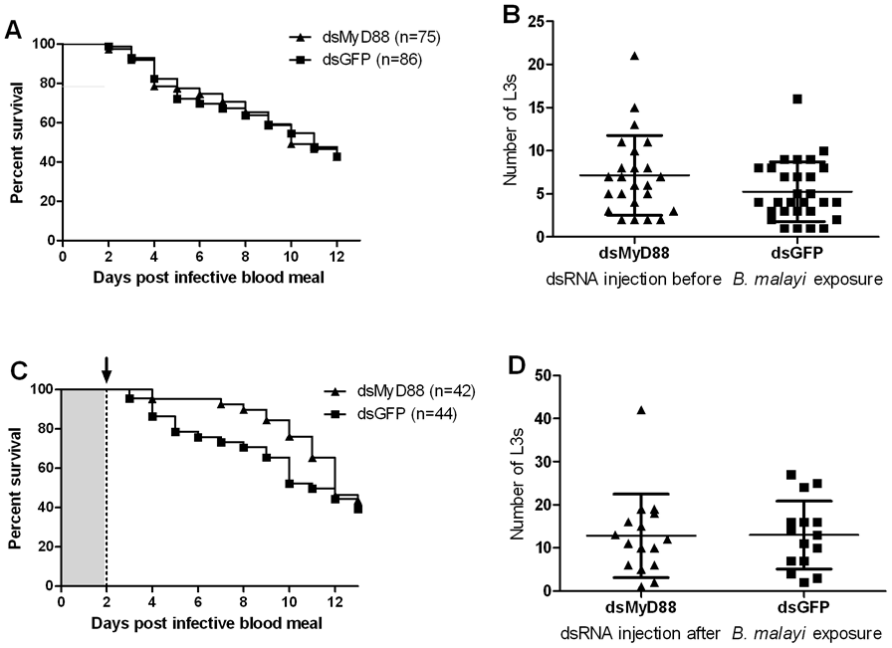
Gene knockdown of *Ae. aegypti* Cactus decreases mosquito survival but does not affect *B. malayi* development (until death of the host). A. Cactus-silenced mosquitoes showed a greater mortality compared to the GFP dsRNA treated controls (P<0.001). B. Mortality of Cactus-silenced mosquitoes fed on uninfected blood displayed a similar mortality rate to those that ingested parasite infected blood (4A). C. Cactus-silenced mosquitoes were dissected at 6 d post blood meal to observe parasite development. D. Gene silencing of *Ae. aegypti* Cactus did not affect the development of *B. malayi* (no difference in L2 mean intensities, P = 0.16). Bars indicate the mean intensity (total number of L3s recovered per infected individual) and standard deviation (D). Log-rank and Mann-Whitney tests were used to compare mosquito mortality curves and parasite mean intensities, respectively. Arrows indicate dsRNA was injected intrathoracically two days following the infective blood meal.

Caspar and MyD88 gene knockdown did not change the mortality rate in either pre- or post-bloodfed dsRNA-injected mosquito groups compared to their respective GFP dsRNA controls (Log-rank tests, P-values ranging from 0.23 to 0.41; see Fig. 3 and 4). In contrast, Cactus gene knockdown, that results in the activation of the Rel1 factor, resulted in a significantly increased mosquito mortality in both pre- and post-blood feeding Cactus dsRNA injected groups (Log-rank test, P<0.001; data not shown). This increased mortality was not related to infection status (Fig. 5A and B).

### Parasite development

There was no significant difference in *B. malayi* mean intensities between Caspar, Cactus or MyD88 silenced mosquitoes compared to the GFP dsRNA injected controls that had fed on the same microfilaremic gerbil. Likewise, there was no difference in prevalence or mean intensity of L3s between Caspar and MyD88 depleted mosquitoes as compared to their respective GFP dsRNA injected controls before (Fig. 3B and 4B) or after (Fig. 3D and 4D) blood feeding. Although knockdown of Cactus increased the mortality rate of *Ae. aegypti*, parasites that were recovered from live mosquitoes at six and 12 d PI had developed normally and there was no difference in the infection prevalence or mean intensity compared to the controls (Figure 5).

## Discussion

In this study, we provide insights into the interactions between filarial worms that cause human LF and their compatible mosquito vectors. As *Brugia* and *Wuchereria* parasites develop, the mosquito experiences a series of insults that include: (1) the penetration of cells and tissues by mf, (2) the consumption of cellular material by developing larvae, and (3) the migration of L3s through the body cavity.

The infection response of mosquitoes is surprisingly diverse during the course of nematode development, as different gene transcripts and regulatory trends were observed in each of the five different developmental time points examined. By infection response we refer to the overall transcriptional and physiological change that occurs in the mosquito as a result of parasite infection, and it includes a vast array of distinct types of responses (e.g., repair, immune, metabolic, reproductive, behavioral, etc.). Overall, the response to filarial worm infection in this compatible system is mainly comprised of molecules involved in cellular signaling, proteolysis, stress response, transcriptional regulation, and repair (see Table S3). And these include several genes that have traditionally been classified as immunity related (Table 1). Very few transcriptional changes were observed until L2s were present, and the most profound transcriptional changes were observed in mosquitoes that harbored infective-stage parasites for 4–5 days. A large proportion of the regulated transcripts represented genes of unknown function (32.9%), and genes that have multiple or diverse functions (40%).

As expected, the transcriptomic profiles of *B. malayi*-infected *Ae. aegypti* are very different than those previously described in *B. malayi*-infected *Armigeres subalbatus* (see Table S4) [27]. In this non-compatible relationship, *B. malayi* development does not occur due to the rapid recognition and melanization of mf in the hemocoel of *Ar. subalbatus* [28]. The different transcriptional changes following infection of mosquitoes that support parasite development and those that do not can provide clues to the molecular mechanisms that determine compatible versus incompatible mosquito-filarial worm associations. Such comparisons have been made between *Cx. pipiens* and *Ae. aegypti* infected with *W. bancrofti* [29], but transcriptional responses that occur in these mosquitoes may not represent genes that are used to deter filarial worm infection in an incompatible system, i.e., it is quite possible that differences in gene transcription of mosquitoes in different genera could represent unique strategies for overcoming damage caused by filarial worms and therefore do not represent anti-filarial worm responses. Similarly, identification of immune-responsive genes activated in response to filarial worm infection does not indicate that the mosquito is/has mounted an immune response against the parasites itself [30]. It is possible that the observed response could be an indirect effect caused by the infection, i.e., mf midgut penetration or muscle cell damage that occurs later in development in compatible mosquitoes. The current study provides transcriptional data from a strain of *Ae. aegypti* that is highly compatible with *B. malayi*, and can help guide the planning of future studies measuring transcriptional changes in a strain of *Ae. aegypti* that does not support the development of *B. malayi*.

The differences in parasite size (Fig. 1) and behavior among developmental stages provide a foundation for discussing the response of *Ae. aegypti* to a successful *B. malayi* infection. In mosquitoes sampled from 1–24 h PI, mf are in the process of penetrating the midgut epithelium and migrating through the hemocoel to penetrate the indirect flight muscle cells. Differentiation to L1s begins as soon as mf become intracellular parasites. At this early stage of infection, transcriptional profiles suggest the presence of *B. malayi* may alter blood digestion/proteolysis (four serine proteases; sterol trafficking), chitin-related interactions (two transcripts contain the chitin-binding Peritrophin-A domain; IPR002557), and immune function (DEF D; AAEL003857).

*B. malayi*-infected mosquitoes sampled at 2–3 d PI harbor L1s, a stage when parasites have a markedly decreased mobility within the indirect flight muscle cells and are in the process of developing a digestive track. The few infection-responsive transcripts (13 genes) during infection with L1s include three down-regulated immunity-related genes: CEC F (AAEL000625), CEC A (AAEL000627), and a hypothetical protein (AAEL003843) which is a putative knottin with an interesting genomic location; just upstream and on the opposite strand from DEF A (AAEL003841). The regulation of cecropin transcripts in response to pathogens is complex [31], and the interpretation of their decreased transcriptional abundance therefore remains speculative.

Mosquitoes sampled at 5–6 d PI contain worms that have molted to L2s. At this stage in development, the parasites are actively ingesting cellular material, have developed a digestive system (with an open mouth but an anus still closed) and have grown four times in length compared to mf. Even though filarial worms remain intracellular until the molt to L3s, these internally damaged cells are likely to provide the necessary stimuli for the mosquito's infection response. Genes involved in cellular signaling (e.g., G-protein coupled receptors, Spaetzle 5, DSCAM) and transcriptional regulation (i.e., changing patterns in transcription factors) were identified as components of the infection response at this time interval (Table S3). Another component of the infection response to L2s is the increased abundance of six cecropin transcripts (Table 1).

At 8–9 d PI, worms have molted to L3s and begun migrating from the thoracic musculature to the mosquito head and proboscis. In *Ae. aegypti*, the onset of severe muscle damage occurs when L3s have exited the infected muscle cells [6]. It has been noted that this muscle damage, and the subsequent damage repair response, also occurs in mosquitoes that are mechanically damaged by external thoracic punctures [32]. Considering these ultrastructural observations by Beckett *et al.* [6], [32]–[34], it is interesting to find that the infection response in this group involved only modest changes in transcript abundance. Of the 26 infection responsive transcripts identified during L3 migration, the majority are induced and putatively involved in stress response (n = 4) and transcriptional regulation (n = 4; see Table S3).

The most profound transcriptomic changes, in response to infection, occurred at 13–14 d PI, an infection stage when mosquitoes have harbored L3s for approximately 4–5 days. Many components of intra- and extracellular signaling pathways (n = 12; see Table S3) were differentially transcribed in these infected mosquitoes, again lending support to the fact that detection and communication of stimuli is key to mounting a response and repairing tissues. L3-infected mosquitoes seem to be responding to tissue damage with 15 genes identified with possible functions in proteolysis and seven insect cuticle protein transcripts in increased abundance. There are multiple sources of tissue damage at this point in infection: (1) the degrading muscle cells that supported the development of the parasites [6], (2) L3 migration throughout the mosquito body cavity [35], and (3) the ability of L3s to penetrate through the cuticular surface [36]. The data also show that infected mosquitoes are responding to the stressful conditions of harboring L3s (9 stress response-related transcripts).

Studies have shown that mosquito behavior can be modified by filarial worm infection, and occurs in an intensity-dependent manner [9]. Comparisons between an earlier study of spontaneous flight behavior changes in *Brugia*-infected *Ae. aegypti* [9] and transcriptional changes seen in the present study could be made for three of our five groups. The estimated mean intensities of *Brugia* infections in mosquitoes collected for transcriptional analysis fit within the categories of low (1–10 parasites) to moderate (11–20 parasites) intensities created by Berry *et al.* [9]. Changes in the transcriptome of *Brugia*-infected mosquitoes are associated with an increase in flight behavior during the time L2s are feeding (Group 3; 5–6 d PI), a marked decrease followed by recovery of flight when L3s emerge and migrate from infected muscle cells (Group 4; 8–9 d PI), and up to a 60% decrease in spontaneous flight activity when mosquitoes harbor *Brugia* L3s (Group 5; 13 d PI). The data from ultrastructural [6], [32]–[34], behavioral [9], and transcriptional observations (presented herein) all support the fact that filarial worm development is not a benign infection to mosquitoes.

Certain mosquito-borne pathogens are known to be controlled by vector immune responses, which are regulated by intracellular signaling pathways, such as Toll and Imd. For example, the Toll and Imd pathways in *An. gambiae* regulates infection with malaria parasites (*Plasmodium berghei* and *P. falciparum*) and is required for antibacterial defenses [26],[37]. Innate immune response in tsetse flies has also been implicated in regulating the intensity of trypanosome infection [38]. An immune response is considered a mechanism by which a host attempts to eliminate or reduce an infection. A host's immune response to parasitism may however not always lead to an elimination of parasites because of the latter's capacity to evade the immune defense mechanisms [39]. Previous studies in our laboratory suggest that LF parasites either elicit an immune response, e.g., melanotic encapsulation, or go undetected and therefore unmolested by an immune response in certain mosquitoes [28],[40]. Although the interactions between these nematodes and the mosquito immune system are mechanistically undescribed, there is potential for LF parasites to evade and/or suppress the mosquito immune system [41]–[44]. In this study, we manipulated the mosquito immune system in an effort to activate immune response pathways to determine what effects, if any, they might have on parasite infection and development.

We used a RNAi–mediated gene knockdown approach to transiently activate the two major immune signaling pathways, Toll and Imd, by targeting their negative regulators, Cactus and Caspar, respectively. Post-transcriptional silencing of these pathway regulators leads to pathway-specific immune responses. Previous studies on the effect of mosquito immune responses on filarial worm development utilized bacterial challenges to activate the immune system [15],[16]. We selected time points for the activation of immune pathways that would specifically target the parasites early in development, i.e., when they might be most vulnerable to the mosquito immune system; when microfilariae migrate to the thoracic musculature and when parasites undergo the first molt (first- to second-stage larvae). The activation of these immune pathways had no detectable effect on *B. malayi* development in *Ae. aegypti* (Fig. 3–5). The lack of an anti-parasite effect as a result of activating Toll and Imd pathways suggest that the parasite limiting mechanism, that was observed in bacterial-challenged *Ae. aegypti*, was not attributed to a Rel1 or Rel2 nuclear translocation. This may imply that the bacteria challenge induced some other defense system, independently of the Toll or Imd pathways, or that the bacteria exerted a direct anti-parasitic effect on the filarial worms.

Infection of *Ae. aegypti* with a compatible filarial parasite, *B. malayi*, resulted in fairly few changes in the mosquito transcriptome; however, these infection responses were diverse and differed vastly between the different infection stages. The majority of these transcriptional infection responses are most likely a reflection of the mosquito's attempt to repair tissue damage resulting from nematode development. We have also shown that removing the inhibitors of Rel1 and Rel2 activation did not affect the permissiveness of this mosquito to *B. malayi* infection. This observation may indicate a resistance, immune evasion and/or suppression stategy(ies) by the parasite, whereby it remains inert to destruction by the mosquito's immune system.

Not all mosquitoes respond the same to LF parasite infection, and differences between natural and artificial systems should be carefully considered. *Aedes aegypti* is a common laboratory vector that has been genetically selected for susceptibility to many pathogens, including *B. malayi* [45], but is not a natural vector of LF parasites [46]. Investigations of flight muscle cell damage caused by developing *B. malayi* in natural and artificial vectors have concluded that tissue damage is more severe in *Ae. aegypti* compared to *Mansonia uniformis*, which is a natural vector [6],[34]. This increased pathology occurs when L3s migrate out of the flight muscle cells, and is reflected by a spike in *Ae. aegypti* mortality [47]. It is apparent that *Ae. aegypti* may utilize different mechanism(s) for surviving infection, and future studies comparing the infection response of natural mosquito-LF parasite systems would allow a better assessment of these differences.

As advancements are made within the field of lymphatic filariasis parasite-host interactions, it will be interesting to compare the infection responses of both the vertebrate and invertebrate hosts. Mf and L3s are the developmental stages transmitted between hosts, and are known to elicit a vertebrate immune response [48]–[50]. The short time interval between L3s escaping the mosquito and infecting the vertebrate host exemplifies the link between the two host environments. For example, the unknown mechanism(s) employed by L3s to suppress the infection response of vertebrates [51] might be functional before L3 escape and may therefore also act on the innate immune response of the mosquito.

## Supporting Information

Table S1 PCR primer sequences.
(0.11 MB RTF)

Table S2 The development of *Brugia malayi* in *Aedes aegypti* (black-eyed, Liverpool) was recorded each time mosquitoes were collected for transcriptional analysis.
(0.07 MB RTF)

Table S3 *Aedes aegypti* transcriptome data. The *Aedes aegypti* response to filarial worm infection is comprised of molecules involved in cellular signaling, proteolysis, stress response, transcriptional regulation, and repair.
(0.08 MB XLS)

Table S4 Comparative analysis to filarial infection responses of a non-compatible mosquito (*Armigeres subalbatus*).
(0.05 MB XLS)

Table S5 *Aedes aegypti* transcriptome data. Log_2_ transformed and non-transformed expression ratio (infected/non-infected) data of the five experimental groups.
(1.38 MB XLS)

Text S1 Validation of microarray gene expression with real-time RT-PCR (RT-qPCR). The expression values (log_2_ ratios) for four genes in three separate time points are plotted against the RT-qPCR expression values. The Pearson's correlation coefficient of 0.889 and the goodness of the fit (R^2^ = 0.790) indicates a high degree of correlation.
(1.19 MB RTF)
