# Supplementary material for: Mosquito Infection Responses to Developing Filarial Worms
Source: PLoS Negl Trop Dis. 2009 Oct 13;3(10):e529. doi: 10.1371/journal.pntd.0000529 (PMC2752998; doi:10.1371/journal.pntd.0000529)
Supplement: Table S1 — PCR primer sequences. (0.11 MB RTF) [file pntd.0000529.s001.rtf]

Table S1.- Primer sequences used in the silencing of genes and validation of microarray data.
					
Primer Name	Primer sequence	 	 		
Ae-SPZ5-F	5'-CGCAGGCAGCTTTGAACAC-3'				
Ae-SPZ5-F	5'-CACTCGCTTGACGTGCATATCTC-3'				
					
Ae-S7-F	5'-GCAGACCACCATTGAACACA-3'				
Ae-S7-R	5'- CCGTTTGGTGAGGGTCTTTA-3'				
					
Ae-Def-F	5'-TGCGCTCCCAGAGGAACT-3'				
Ae-Def-R	5'-CACAGCCGCTTGGTAAGATTC-3'				
					
Ae-Cec-F	5'-TCACAAAGTTATTTCTCCTGATCG-3'				
Ae-Cec-R	5'-GCTTTAGCCCCAGCTACAAC-3'				
					
Ae-CLIP-F	5'-CGCTGCTCACTGCATCAAAC-3'				
Ae-CLIP-R	5'-CTCGCAATCTACTTCCTGTCCTTT-3'				
					
dsCact-F	5'-TAATACGACTCACTATAGGG CGAGTCAACAGAACCCGAGCAG-3'			
dsCact-R	5'-TAATACGACTCACTATAGGG TGGCCCGTCAGCACCGAAAG-3'			
					
dsCact-eff-F	5'-CCAGACACCGCTCCACAT-3'				
dsCact-eff-R	5'-TCCAGCAGGAAGTTCACCAA-3'				
					
dsMyD88-F	5'-TAATACGACTCACTATAGGGGGCGATTGGTGGTTGTTATT-3'			
dsMyD88-R	5'-TAATACGACTCACTATAGGGTTGAGCGCATTGCTAACATC-3'			
					
dsMyD88-eff-F	5'-CATCCCATTCAGTTTCTCAGC-3'				
dsMyD88-eff-R	5'-ACCGGTTGGAAGTTCTGATG-3'				
					
dsCaspar-F	5'-TAATACGACTCACTATAGGG GGAAGCAGATCGAGCCAAGCAG-3'		
dsCaspar-R	5'-TAATACGACTCACTATAGGG GCATTGAGCCGCCTGGTGTC-3'			
					
dsCaspar-eff-F	5'-GAATCCGAGCGAGCCGATGC-3'				
dsCaspar-eff-R	5'-CGTAGTCCAGCGTTGTGAGGTC-3'	 	 		
					
