# Supplementary material for: Mosquito Infection Responses to Developing Filarial Worms
Source: PLoS Negl Trop Dis. 2009 Oct 13;3(10):e529. doi: 10.1371/journal.pntd.0000529 (PMC2752998; doi:10.1371/journal.pntd.0000529)
Supplement: Table S2 — The development of Brugia malayi in Aedes aegypti (black-eyed, Liverpool) was recorded each time mosquitoes were collected for transcriptional analysis. (0.07 MB RTF) [file pntd.0000529.s002.rtf]

Table S2.  The development of Brugia malayi in Aedes aegypti (black-eyed, Liverpool) was recorded each time mosquitoes were collected for transcriptional analysis.
Groupa	Time post feeding	Percentage of mosquitoes harboring worms (total dissected)	Developmental stage of Brugia malayib	Total worms	
			Microfilariae	L1	L2	L3		
1	1 hr	 93% (15)	100% (233)				233	
1	6 hr	100% (15)	99.7% (395)	0.3% (1)			396	
1	12 hr	 80% (15)	84.6% (203)	15.4% (37)			240	
1	24 hr	 93% (15)	61.7% (291)	38.3% (181)			472	
2	2 d	 87% (15)	40.9% (74)	59.1% (107)			181	
2	3 d	100% (15)	0.9% (1)	99.1% (109)			110	
3	5 d	100% (15)			100% (120)		120	
3	6 d	100% (15)			100% (179)		179	
4	8 d	100% (15)			41.1% (74)	58.9% (106)	180	
4	9 d	 93% (15)			10.4% (13)	89.6% (112)	125	
5	13 d	 73% (15)				100% (93)	93	
5	14 d	 80% (15)				100% (72)	72	
aMosquito RNA was combined into 5 groups for gene expression analysis based on parasite development within the vector.
bThe percentage of total worms at specified developmental stage (number observed).
