# Supplementary material for: Mosquito Infection Responses to Developing Filarial Worms
Source: PLoS Negl Trop Dis. 2009 Oct 13;3(10):e529. doi: 10.1371/journal.pntd.0000529 (PMC2752998; doi:10.1371/journal.pntd.0000529)
Supplement: Text S1 — Validation of microarray gene expression with real-time RT-PCR (RT-qPCR). The expression values (log2 ratios) for four genes in three separate time points are plotted against the RT-qPCR expression values. The Pearson's correlation coefficient of 0.889 and the goodness of the fit (R2 = 0.790) indicates a high degree of correlation. (1.19 MB RTF) [file pntd.0000529.s006.rtf]

Text S1


Validation of microarray gene expression with real-time RT-PCR (RT-qPCR). The expression values (log2 ratios) for four genes in three separate time points are plotted against the RT-qPCR expression values. The Pearson's correlation coefficient  of 0.889 and the goodness of the fit (R2 = 0.790) indicates a high degree of correlation.


Correlation of log2 ratios from microarray expression data with log2 RT-qPCR expression values.

Group	Gene Name		Log2 ratio	
		Vectorbase 
Accession number	RT-qPCR	Microarray	
Group 2	Cec	AAEL000625-RA	-5.64	-1.00	
	Def	AAEL003857-RA	-3.46	-0.12	
	Spz5	AAEL001929-RA	0.34	0.21	
	CLIPSP	AAEL005060-RA	-0.56	-0.12	
					
Group 3	Cec	AAEL000611-RA	1.63	1.60	
	Cec	AAEL000598-RA	3.32	1.50	
	Cec	AAEL015515-RA	2.54	1.40	
	Spz5	AAEL001929-RA	0.55	0.76	
	CLIPSP	AAEL005060-RA	0.38	0.20	
	CLIPSP	AAEL005431-RA	0.93	0.23	
					
Group 5	CLIPSP	AAEL005060-RA	1.81	1.2	
	CLIPSP	AAEL007511-RA	1.81	1.1	
